# Orofacial Manifestations and Temporomandibular Disorders of Systemic Scleroderma: An Observational Study

**DOI:** 10.3390/ijms17071189

**Published:** 2016-07-22

**Authors:** Vito Crincoli, Laura Fatone, Margherita Fanelli, Rossana Patricia Rotolo, Angela Chialà, Gianfranco Favia, Giovanni Lapadula

**Affiliations:** 1 Complex Operating Unit of Odontostomatology, Interdisciplinary Department of Medicine, “Aldo Moro” University of Bari., Piazza Giulio Cesare 11, 70124 Bari, Italy; laura.fatone@gmail.com (L.F.); rossanarotolo@gmail.com (R.P.R.); gianfranco.favia@uniba.it (G.F.); 2 Complex Operating Unit of Nuclear Medicine—Medical Statistics, Interdisciplinary Department of Medicine, “Aldo Moro” University of Bari., Piazza Giulio Cesare 11, 70124 Bari, Italy; margherita.fanelli@uniba.it; 3 Complex Operating Unit of Rheumatology, Interdisciplinary Department of Medicine, “Aldo Moro” University of Bari., Piazza Giulio Cesare 11, 70124 Bari, Italy; angelachiala@libero.it (A.C.); giovanni.lapadula@uniba.it (G.L.)

**Keywords:** scleroderma, oral manifestation, temporomandibular disorders

## Abstract

Scleroderma is a disorder involving oral and facial tissues, with skin hardening, thin lips, deep wrinkles, xerostomia, tongue rigidity, and microstomia. The aim of this study was to investigate the prevalence of oral manifestations and temporomandibular disorders (TMD) in Systemic Sclerosis (SSc) patients compared with healthy people. Eighty patients (6 men, 74 women) fulfilling ACR/EULAR SSc Criteria were enrolled. A randomly selected group of 80 patients, matched by sex and age served as control group. The examination for TMD signs and symptoms was based on the standardized Research Diagnostic Criteria for Temporomandibular Disorders (RDC/TMD) through a questionnaire and clinical examination. SSc patients complained more frequently (78.8%) of oral symptoms (Xerostomia, dysgeusia, dysphagia and stomatodynia) than controls (28.7%) (χ^2^ = 40.23 *p* = 0.001). TMD symptoms (muscle pain on chewing, difficulty in mouth opening, headaches) were complained by 92.5% of SSc patients and by 76.2% of controls (χ^2^ = 8.012 *p* = 0.005). At the clinical examination, 85% of SSc patients showed restricted opening versus 20.0% of controls (χ^2^ = 67.77 *p* = 0.001), 81.2% of SSc showed reduced right lateral excursion versus 50% of controls (χ^2^ = 17.316 *p* = 0.001); 73.8% of SSc showed limited left lateral excursion versus 53.8% of controls (χ^2^ = 6.924 *p* = 0.009); and 73.8% of SSc had narrow protrusion versus 56.2% of controls (χ^2^ = 5.385 *p* = 0.02).

## 1. Introduction

Scleroderma is a disorder of the connective tissue characterized by fibrosis of the skin, vascular abnormalities, and presence of auto-antibodies. It is a rare disease with a prevalence from 8 up to 30/100,000 inhabitants. Women are affected more frequently than men are, with a preponderance of 4:1 [1]. The etiological agent is still unknown, even if previous findings evidence that the fibrotic process could be activated by lesional fibroblast activation [2,3,4].

It can be divided into two major groups: localized scleroderma /morphea and systemic sclerosis (SSc). The former can be classified into four main subtypes (plaque, bullous, linear, and deep) according to morphological and clinical presentation and is traditionally considered as a skin-limited disease whose systemic involvement is rare. The latter is classified into three categories including limited cutaneous SSc, diffuse cutaneous SSc, and overlap syndrome. SSc differs from localized scleroderma because it is accompanied by Raynaud’s phenomenon, acrosclerosis, and internal organ involvement [5,6]. The extracutaneous manifestations include gastrointestinal tract, heart, lungs and kidneys: esophageal dysmotility, restrictive pulmonary disease, pulmonary hypertension, arthralgia, myopathy, myocardiopathy, and progressive renal insufficiency can be observed [7]. Raynaud’s phenomenon, i.e., vasospasm of fingers which results in change in color of fingertips as a response to cold or emotion, is often the initial presenting complaint occurring in more than 95% of SSc patients. Limited SSc is characterized by symmetric and progressive skin fibrosis of the distal upper extremities (fingers) whereas diffuse SSc patients often develop widespread, symmetrical fibrosis involving the proximal and distal extremities, trunk, and face [5,6,8].

**Figure 1:**
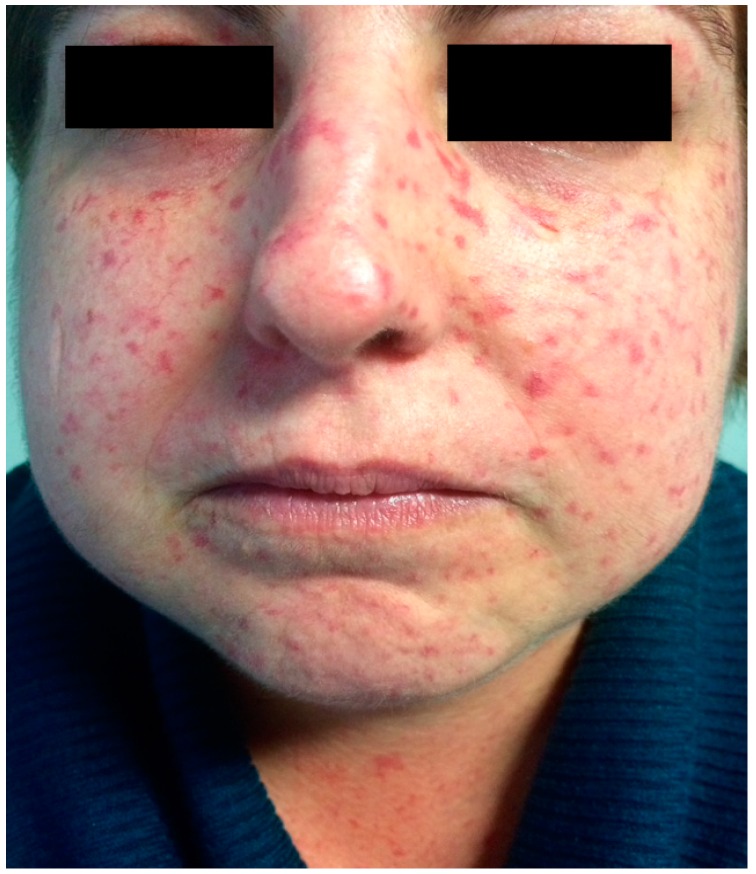
Presence of telangiectasia.

Oral and facial tissues are often affected, presenting characteristic features. Most clinical manifestations begin with tongue rigidity and facial skin hardening, leading to a classic mask-like face, white thin lips, a sharp nose, and deep wrinkles [9,10]. Telangiectasia of facial skin and oral mucous membrane are produced from the vasospasm of the small vessels (Figure 1).

SSc patients also manifest periodontal involvement, demonstrated by increased pocket depths and gingival scores [11]. The study by Pischon et al. demonstrates higher periodontal clinical attachment loss in SSc patients if compared with healthy people, indicating a possible relationship between SSc and periodontitis [12]. The etiology of periodontal disease in SSc is unclear and several factors could contribute to periodontal involvement. An obliterative microvasculopathy was suggested [11]. Wood et al. [13] indicated a relation with the reduced vascularity, with resulting tissue ischemia in scleroderma individuals. An association of reduced mouth opening and presence of periodontal disease is supposed [13] but not confirmed by Chu et al. [14]. Xerostomia is a common finding with scleroderma patients, occurring as a result of fibrosis of the salivary glands. Dry-mouth symptoms can cause a high caries rate and an increased incidence of Candida infections [15]. Xerostomia could promote dental plaque accumulation and a reduced manual dexterity could be a cofactor in periodontal involvement in SSc patients.

The tongue can also become rigid so that speech and swallowing become difficult [16].

**Figure 2:**
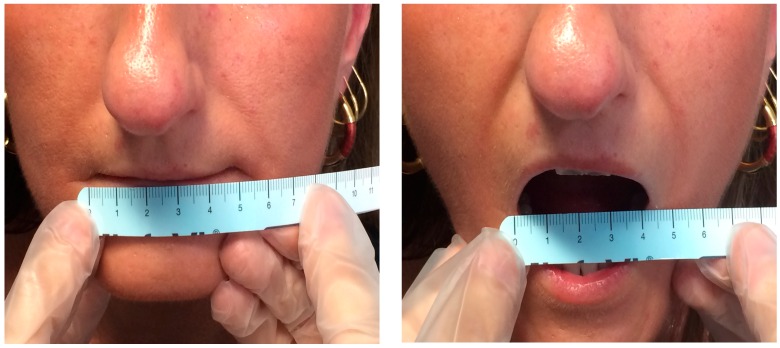
SSc patient. Microstomia has been measured as the distance between the lip commissures.

The main oral manifestation of SSc is microstomia (reduction of the oral opening), due to the sclerosis of perioral soft tissue (Figure 2). Clinically, it may impair social relationships, mastication, reduction of the mandibular movements, and proper oral hygiene. Consequently, a higher incidence of oral diseases such as caries, periodontal diseases or other type of oral infections is detected [17] and dental treatment could be more difficult or sometimes impracticable due to limited mouth opening [14,18].

Mandibular bone resorption is more often observed in patients with diffuse cutaneous SSc and causes marked facial sclerotic involvement and limitation in mouth opening [19,20]. Systematic radiographic screening of different groups of SSc patients showed a resorption incidence of the mandible of 20%–33% of the examined mandibles [19]. In 1959, Taveras noted radiographic evidence of bone resorption of the angles of the mandible and similar areas of resorption have been reported to affect the coronoid processes and the zygomatic arches at the sites of the muscle attachments. This was explained by muscle contracture secondary to fibrotic changes [21]. However, the current hypothesis suggests that bone resorption could be caused by a multifactorial process including microvasculopathy and pressure ischaemia, secondary to thickening skin and muscle atrophy. Moreover, atrophic and fibrotic modifications of the synovia may also influence the temporomandibular joint (TMJ) involvement in course of SSc [22].

**Figure 3:**
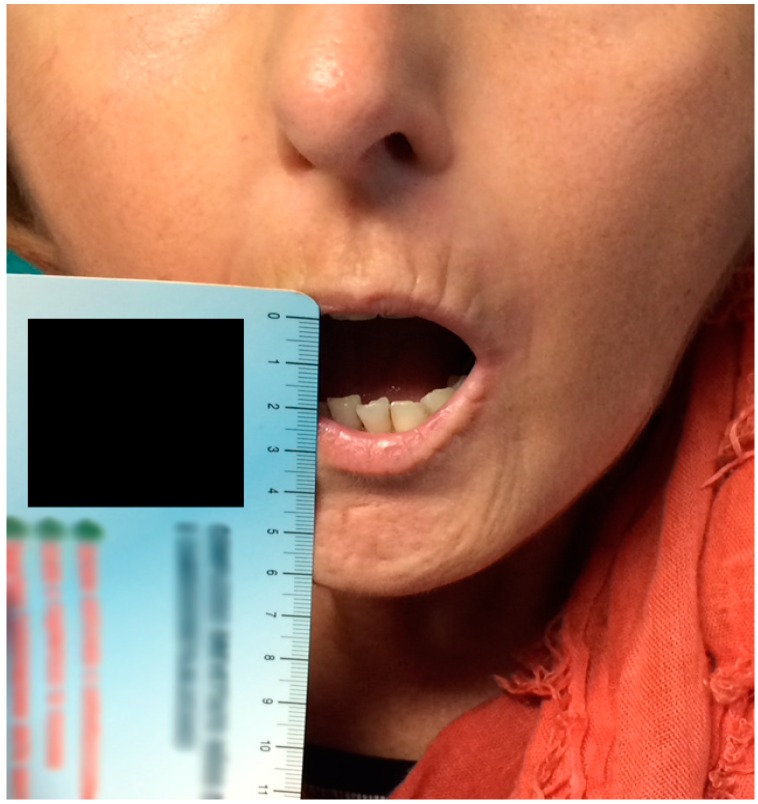
Maximum active opening: 19 mm, negative end-feel. Pronounced labial wrinkles, sharp nose.

Previous studies investigated the difficulty in mandibular movements in SSc patients. Nagy observed that the interincisal distance was significantly decreased in systemic sclerosis patients when compared with healthy controls [23] (Figure 3).

Marmary found that the majority of SSc patients revealed limitation in mouth opening [24].

Only a few studies reported a complete analysis of signs and symptoms of temporomandibular disorders (TMD) [18,25].

The aim of this study was to investigate the prevalence of both oral symptoms and manifestations and TMD symptoms (TMDs) and signs in SSc patients compared with healthy people, thus giving a complete survey of facial involvement in course of scleroderma.

## 2. Results

### 2.1. Characteristics of SSc Patients and Controls

Patients’ age ranged between 24 and 80 years, 92.5% were female and 7.5% were male in both groups. The two groups, matched for age and sex, results were similar for sociodemographic aspects, except occupation (χ^2^ = 11.036 *p* = 0.026). Among SSc patients housewives were prominent, while among controls office workers were prominent (Table 1).

The prevalent form of disease was the diffuse one, found in the 82.5% of SSc patients, the age at diagnosis varied between 8 and 74 years (mean = 44 years, SD = 14.24) with a disease mean duration of 12 years (SD = 8.39); 60% of patients had the pathology for over 10 years (Table 2).

Clinical characteristics of SSc patients and controls are reported in Table 3.

The principal drugs for SSc patients and controls are reported in Table 4.

### 2.2. SSc Oral Symptoms

The assessment of referred SSc oral symptoms, obtained from written responses to the questionnaire, revealed that the patients with SSc complained more frequently than controls: 78.8% of the SSc patients complained of one or more symptoms compared to 28.7% of the controls (χ^2^ = 40.23 *p* < 0.001). Statistically significant differences were found between two groups for each oral symptom considered as listed in Table 5.

### 2.3. TMD Symptoms

The assessment of TMDs showed that 92.5% of the patients with SSc and 75.0% of the healthy controls complained of one or more symptoms. Statistically significant differences were found between the two groups (χ^2^ = 8.012 *p* = 0.005).

In the SSc group, muscle pain on chewing, the sensation of a stuck or locked jaw, and arthralgia were the most commonly reported symptoms. In the control group tenderness or stiffness in the neck and shoulders were the major complaints.

Myofascial pain (MP) evoked by palpation was detected in 91.2% SSc patients and 83.8% of controls, revealing no significant difference between the two groups (χ^2^ = 2.057 *p* = 0.151). Data collected for each muscle couple were reported in Table 6.

### 2.4. SSc Oral Signs

At the clinical examination, oral signs correlated with SSc were more frequent in the study group than in the control one; 98.8% of SSc patients have at least one oral sign compared with 45% of controls (χ^2^ = 54.54 *p* < 0.001). Statistically significant differences were observed for each sign except for fibrous tongue. Table 7 lists the main findings collected from the oral examination.

### 2.5. TMJ Signs

At the clinical examination, restricted movements (RM) were more frequent in the SSc group than in the healthy controls. In particular, 85% of SSc patients showed restricted opening versus 20.0% of controls (χ^2^ = 67.77 *p* < 0.001); 81.2% of SSc showed reduced right lateral excursion versus 50% of controls (χ^2^ = 17.316 *p* < 0.001); 73.8% of SSc showed limited left lateral excursion versus 53.8% of controls (χ^2^ = 6.924 *p* = 0.009); and 73.8% of SSc had narrow protrusion versus 56.2% of controls (χ^2^ = 5.385 *p* = 0.02).

Measurement data related to movements were reported in Table 8.

Evidence of bruxism was observed in 81.2% of patients with SSc and 75% of controls, with no significant difference between the two groups (χ^2^ = 0.914 *p* = 0.339).

The prevalence of opening derangement (OD) was higher in controls (53.8%) than in SSc patients (43.8%). although this difference was not statistically significant (χ^2^ = 1.6 *p* = 0.206). Finally, no differences were recorded for sounds of temporomandibular joint (TMJs) in the two groups: this sign was observed in 50% of SSc patients and 50% of controls (χ^2^ = 0 *p* = 1).

## 3. Discussion

To our knowledge, the present study is the only report that analyzed and evaluated the prevalence of sign and symptoms of both mucosal damage and temporomandibular dysfunction in SSc compared with healthy controls with a cohort size larger than other studies investigating TMD and SSc (80 SSc patients versus 35 patients in Ferreira et al. [18], 14 SSc patients in Aliko et al. [22], and 68 SSc patients in Matarese et al. [25]).

About sociodemographic characteristics, unemployed and housewives were most represented in SSc patients, while office workers were most represented among controls (Table 1). These data could be explained with a progressive physical disability in SSc group, leading to a severe impairment of patients’ quality of life to varying degrees, with troubles in catching and even in writing. Also psychological aspects, such as self non-acceptance, can damage social life.

An interesting finding in patients’ history (Table 3) is osteoporosis, affecting 19 (23.8%) SSc patients versus 1 (1.2%) of control group. Consequently, a higher rate of SSc patients (17.1%) takes bisphosphonates (Table 4). However, a long-term antiresorptive therapy could cause, as a side effect, medication-related osteonecrosis of the jaws (MRONJ) after oral surgery. Therefore, in daily routine, dentists should consider SSc patients a high potential risk category.

The SSc group showed more symptoms and signs of mucosal and temporomandibular joint (TMJ) involvement compared with the control group.

Sicca symptoms are thought to be a frequent complaint associated with SSc, although little is known about how often they occur. Xerostomia was often complained by SSc patients recruited for the present study (42%), showing a higher prevalence if compared with findings by Bajraktari et al., who recorded this oral manifestation in 32% of SSc patients [26] and with findings by Weisman et al., who detected features of sicca syndrome in 12% of SSc patients [27]. Wood recorded xerostomia in 70% of the SSc patients and associated it with an increased frequency of dental caries [13].

According to Avouac et al. [28], the main cause of sicca syndrome in SSc appears to be glandular fibrosis, rather than lymphocytic sialadenitis associated with Sjögren’s syndrome: on 78 biopsy specimens obtained from patients with subjective symptoms of xerostomia, 23% of these cases were explained by Sjögren’s syndrome and 58% were explained by glandular fibrosis. According to Vincent et al., xerostomia was considered more discomforting (mean VAS = 3.8) than decreased mouth opening (mean VAS = 2.6) and was significantly associated with the limited cutaneous form [29].

Dysphagia was reported by 51.3% of SSc patients collected in this study and it was the most frequent otolaryngologic symptom complained by SSc patients compared with controls. As assessed in a study by Weisman et al., where 39% of SSc patients showed this symptom, radiographically detected esophageal abnormalities could be suggestive of dysphagia, though they did not always record this complaint even with an abnormal esophagram. It could be caused by a loss of contractile power of the esophagus, alterations of esophageal mucosa, abnormalities of the tongue and pharynx [27].

The present study, gathering information on both remote and recent anamnesis, detects a higher prevalence of TMDs in the SSc group (92.5%) compared to the respective outcomes in healthy patients (75.0%). These data differ from ones by Matarese et al. [25] that recorded TMDs in 74.1% of their 27 SSc patients.

An etiological hypothesis of the high prevalence of TMD in SSc could insist in the disease duration. In this work, in fact, in the 60% of SSc group the disease diagnosis dates back more than 10 years (Table 2).

MP evoked by palpation was reported by 92.5% of SSc patients in the present work, with a remarkably higher prevalence if compared with Matarese findings (55.6%) [25]. A mild, progressive myositis can lead to different degrees of atrophy and fibrotic muscle retraction when an inflammatory reaction occurs: this could explain the increased frequency of complaints in the SSc group [30].

TMJs were detected in the same proportion both in SSc group (45%) and controls (50%). Different findings were obtained by Matarese et al. [24], who recorded TMJs in 63% of SSc patients and 7.1% of their controls. Such a difference could be explained with a diverse selection of controls. In the present study, the sample was selected from people attending a dental clinic, who would report TMD more frequently than the general population.

The main expected finding was a more severe restriction on mandibular movements in SSc patients than in controls. In this work, RM were detected in 80% of SSc group, in accordance to Marmary’s results [18]. Jaw mobility score was recorded by Ferreira et al.: they observed that none of the SSc patients showed normal width in jaw movements and in particular 77.1% of SSc patients had a severe jaw mobility impairment (all measures were lower than mean values) [18]. On the other side, in a work by Weisman, only 28% of patients had altered kinematics [27]. In this last case, this parameter was recorded as a referred symptom and not as a sign detected by a clinician.

In the present study, maximum opening was considered as a mean value and compared with the healthy group. In the SSc group, maximum mouth opening was assessed ad 33.29 ± 7.97 mm and it was a high value if compared with findings by Matarese, where maximum mouth mean value was found to be 20.11 ± 2.6 mm. Also left (5.87 ± 3.61 mm) and right (5.67 ± 3.14 mm) laterotrusion were recorded as wider movements if compared with Matarese findings (respectively 2.55 ± 0.5 mm and 2.48 ± 0.5 mm) [25]. Differently from other rheumatological diseases, i.e., rheumatoid or psoriatic arthritis, where the TMDs were associated with joint inflammation, the dysfunction of the masticatory system and the severe restriction of jaw mobility in SSc patients is likely related with the progressive reduction and loss of skin elasticity and increase in skin thickness, because of collagen accumulation, i.e. fibrosis of lips, skin, and subcutaneous tissues [18,31,32]. Although arthropathy in SSc is almost common, it was not possible to determine whether the present findings could be ascribable to TMJ involvement or to lips, skin, and subcutaneous tissues fibrosis [22,33]. Microstomia was recorded in 80% of the SSc group in this work, a higher prevalence if compared with Bajraktari et al.’s [26] findings who found “small mouth” in 52% of SSc patients. According to Maddali [34] the progression of microstomia may be reversed by means of mouth-stretching and oral augmentation, even if Yuen did not assess significant enhancement of mouth opening after six months of orofacial exercise program intervention for SSc patients maybe because of the low exercise adherence rate, insufficient frequencies, repetitions, and durations of exercises [35].

## 4. Materials and Methods

This study was conducted from September 2015 to March 2016 at the School of Dentistry and the Department of Rheumatology, University of Bari, Italy, in accordance with the provisions of the Declaration of Helsinki. Ethical approval and informed consent from each human subject were obtained.

Eighty patients (6 men, 74 women) fulfilling ACR/EULAR SSc Criteria were enrolled in the study group [36]. A group of 80 patients matched by sex and age (6 men, 74 women), randomly selected among those presenting at the Dental Clinic, served as control group.

Inclusion criteria were: (i) over 18 years of age; (ii) Caucasian ethnic origin. According to their medical histories, patients were excluded if they had motor impairments, neurological disorders, or head, oral, or neck cancer. In addition, patients receiving or having undergone orthodontic treatment or maxillofacial surgery were not selected [31,37,38].

Patients age ranged between 24 and 80 years, with a mean age of 56.28 (SD = 12.96) years in the SSc group, and 56.45 years (SD = 13.05) in the controls.

RDC/TMD were applied to collect data [37]. Symptoms and signs in the whole SSc group were recorded by a single experienced practitioner through an anamnestic questionnaire and clinical examination, finally compared with those in the control population.

### 4.1. Patients History

#### 4.1.1. SSc: Oral Symptoms

They were recorded through a questionnaire investigating the presence or the absence of the following complaint and collected as categorical data:

(i) *Xerostomia*: it is considered as the complaint of dry mouth associated to discomfort while eating, speaking, swallowing, and wearing dentures. The mucosa may appear dry and sticky with stringy or foamy saliva [39].
(ii) *Dysgeusia*: it is defined as a distorted gustatory perception. The diagnosis of dysgeusia was based on the patients’ reports where gustatory stimuli are perceived as bitter, sour, or metallic [40].
(iii) *Dysphagia:* it is defined as difficulty in swallowing food (semi-solid or solid), liquid, or both. It was evaluated through swallowing trials using a variety of texture-modified liquids and solids [41].
(iv) *Stomatodynia*: a burning sensation in the mouth, including hurtful sensation or pain, in association with a clinically normal oral mucosa [42].

#### 4.1.2. TMD Symptoms

*TMDs:* Patients’ complaints , collected as categorical data (presence or absence of TMDs), were recorded through a questionnaire investigating masticatory muscle pain at rest and on chewing, neck and shoulders stiffness, TMJ arthralgia, a feeling of locked jaw, and migraines and headaches [31].

Other, less common symptoms, such as dizziness, earache, and tinnitus were also considered [43]. Patients were also asked if TMDs occurred in relation to their self-perceived disease severity, and if they had occurred once or periodically since the disease was diagnosed.

*MP*: it was accurately evaluated. While palpation does not elicit sensations of tenderness or pain in healthy muscles, an ache may be provoked by compression of damaged muscle tissue. The following masticatory muscles were palpated bilaterally: anterior, medial, and posterior temporalis muscles, masseter muscle, medial pterygoid muscle, lateral pterygoid muscle with its superior and inferior head, digastric (anterior and posterior belly) muscle, mylohyoid and sternocleidomastoid muscles. Palpation was performed applying soft but firm pressure to the muscle, mainly with the palmar surface of the thumb and of the index finger.

### 4.2. Clinical Examination

#### 4.2.1. SSc: Oral Signs

*Microstomia*: it is characterized by a reduction of the mouth opening. The values were obtained by measuring the distance between the lip commissures. When, in maximum mouth opening, this distance was lower than the sum of mesio-distal diameters of the six upper frontal teeth, the patient was considered microstomic [44].

*Oral ulcers*: presence of roundish or oval sores was detected inside the mouth.

*Petechiae*: presence of red or brown pinpoint lesions not blanching on pressure was detected in the oral mucosa [45].

*Gingival recession*: recession is considered as a retraction of the gingival margin from the dental crown. Clinical attachment level was recorded through a probe.

*Fibrous tongue*: lingual fibrotic induration and its consequent reduction of mobility was found.

#### 4.2.2. TMJ Signs

*TMJs:* they were assessed by palpation on each side separately on mandible movement. Clicking is considered as a clean, loud jaw joint noise of short duration, while crepitation as multiple, creaking, gravel-like sounds [31].

*Bruxism:* it is a parafunctional jaw-muscle activity, characterized by grinding and clenching of the teeth; it can occur during sleep or when awake [46]. It becomes pathological when associated with

(i) myalgia, caused by prolonged vasoconstriction and accumulation of catabolites in the muscle tissue [47].
(ii) wear facets, with loss of vertical dimension of occlusion.

*OD:* in a balanced masticatory system, the opening trajectory taken by the mandible midline is straight. Alterations of the mandibular kinematics are classified as:

(i) deviation: any shift of the mandibular midline on the frontal occlusal plane during opening, that disappears with continued opening (a return to midline);
(ii) deflection: any shift of the midline to one side that increases with opening and persists at maximum opening [48].

*RM*: They were classified as

(i) reduced opening: in a healthy system, the mouth opens by between 53 and 58 mm. Taking into account overbite [49], a restricted mandibular opening is considered to be any distance of <40 mm.
(ii) right and left lateral shifts, measured from upper to lower midline, were recorded when lower than 8 mm.
(iii) mandibular forward displacement was recorded when lower than 7 mm [50].

### 4.3. Statistical Analysis

The sample size was calculated by expecting the presence of one or more oral symptoms in 60% of the SSc patients and in 30% of controls. The sample size required for an α = 0.01 and 90% power meant 80 patients in each group.

Categorical data were expressed as number and percentage and comparisons between SSc and control patients were performed using chi-squared (χ^2^) test (with Yates’ correction when necessary). Quantitative data were expressed as mean and standard deviation, *t* Student for unpaired samples was employed to compare SSc patients and control patients. In all comparisons, a *p* value ≤0.05 was considered statistically significant. SPSS version 20.0 was used for analysis of data.

## 5. Conclusions

In this observational study, a statistically significant increase of oral and TMD symptoms was consistently detected in SSc subjects. Therefore, in addition to dermatological and rheumatological implications, scleroderma seems to play a role in TMJ disorders, causing an increase in orofacial pain and an altered chewing function.

## Figures and Tables

**Table 1:** Sociodemographic characteristics of SSc patients and controls.

Sociodemographic Characteristics	SSc	Controls	Test	*p* Value
Age, mean ± SD	56.28 ± 12.96	56.45 ± 13.05		
Sex, *n* (%)				
male	6 (7.5%)	6 (7.5%)		
female	74 (92.5%)	74 (92.5%)		
Educational degree, *n* (%)				
primary	17 (21.2%)	9 (11.2%)	χ^2^ = 6.691	0.082
secondary	26 (32.5%)	19 (23.8%)		
high	31 (38.8%)	40 (50%)		
academic	6 (7.5%)	12 (15%)		
Occupation, *n* (%)				
housewife	31 (38.8%)	24 (30%)	χ^2^ = 11.036	0.026
retired	22 (27.5%)	18 (22.5%)		
office worker	19 (23.8%)	34 (42.5%)		
self employed	1 (1.2%)	3 (3.8%)		
not employed	7 (8.7%)	1 (1.2%)		
Marital status, *n* (%)				
married	56 (70%)	63 (78.7%)	χ^2^ = 5.271	0.153
widower	5 (6.2%)	8 (10%)		
single	17 (21.3%)	7 (8.8%)		
divorced	2 (2.5%)	2 (2.5%)		

**Table 2:** Form of disease of SSc patients.

Form of Disease		Type of SSc, *n* (%)	Mean ± SD (Range)
Diffuse		66 (82.5%)	-
Limited		14 (17.5%)	-
Age at diagnosis		-	44 ± 14.24 (8–74)
Disease duration (class of years)	<5 years	21 (26.2%)	-
	5–10 years	11 (13.8%)	-
	>10 years	48 (60%)	-
Disease duration		-	12 ± 8.39 (1–50)

**Table 3:** Clinical characteristics of SSc patients and controls.

Clinical Characteristics	SSc	Controls	Test	*p* Value
Cardiopathy	24 (30%)	3 (3.8%)	χ^2^ = 19.649	<0.001
Diabetes mellitus	5 (6.2%)	1 (1.2%)	χ^2^ = 2.771	0.096
Digital ulcers	16 (20%)	0	Fisher exact test	<0.001
Esophageal disease	26 (32.5%)	2 (2.5%)	χ^2^ = 2..935	<0.001
Gastritis	6 (7.5%)	2 (2.5%)	χ^2^ = 2.105	0.147
Hypertyension	9 (11.2%)	16 (20%)	χ^2^ = 2.323	0.127
Hypovitaminosis	11 (13.8%)	2 (2.5%)	χ^2^ = 6.782	0.009
Interstitial lung disease	27 (33.8%)	0	Fisher exact test	<0.001
Osteoporosis	19 (23.8%)	1 (1.2%)	Fisher exact test	<0.001
Presence of removable prosthesis	10 (12.5%)	15 (18.8%)	χ^2^ = 1.185	0.276
Raynaud syndrome	45 (56.2%)	0	Fisher exact test	<0.001
Thyroid disease	16 (20%)	14 (17.5%)	χ^2^ = 0.164	0.685

**Table 4:** SSc patients and controls’ drugs.

Drugs	SSc	Controls	Test	*p* Value
ACE inhibitors	7 (9.2%)	2 (2.5%)	χ^2^ = 2.112	0.146
Acetylsalicylic acid	19 (25%)	3 (3.8%)	χ^2^ = 14.528	<0.001
Angiotensin II receptor antagonist	5 (6.6%)	0	Fisher exact test	0.026
Antacids	57 (75%)	4 (5%)	χ^2^ = 80.199	<0.001
Beta blockers	4 (5.3%)	19 (23.8%)	χ^2^ = 10.597	0.001
Bisphosphonate	13 (17.1%)	1	Fisher exact test	<0.001
Calcium channel blockers	34 (44.7%)	1 (1.2%)	Fisher exact test	<0.001
Cholecalciferol	43 (56.6%)	4 (5%)	Fisher exact test	<0.001
Digitalis glycosides	2 (2.6%)	0	Fisher exact test	0.236
Diuretics	14 (18.4%)	4 (5%)	χ^2^ = 6.89	0.009
Endothelin receptor antagonists	39 (51.3%)	0	Fisher exact test	<0.001
Iloprost	71 (88.8%)	0	Fisher exact test	<0.001
Immunosuppressant	41 (53.9%)	0	Fisher exact test	<0.001
Lipid-lowering drugs	11 (14.5%)	10 (12.5%)	χ^2^ = 0.130	0.718
Nitrate drugs	4 (5.3%)	0	Fisher exact test	<0.001
Nonsteroidal anti-inflammatory drugs	4 (5.3%)	0	Fisher exact test	0.054
Prokinetic drugs	35 (46.1%)	0	Fisher exact test	<0.001
Thyroxine	10 (13.2%)	15 (18.8%)	χ^2^ = 0.906	0.341
Ursodeoxycholic acid	9 (11.8%)	0	Fisher exact test	0.001

**Table 5:** Subjective complaints of oral discomfort in SSC patients and controls.

Oral Symptoms	SSc	Controls	χ^2^	*p* Value
Xerostomia	42 (52.5%)	17 (21.3%)	16.78	<0.001
Dysgeusia	27 (33.8%)	4 (5.0%)	19.36	<0.001
Dysphagia	41 (51.3%)	3 (3.8%)	42.91	<0.001
Stomatodynia	23 (28.8%)	4 (5.0%)	14.44	<0.001

**Table 6:** Myofascial pain in SSC patients and controls.

Muscle	SSc	Controls	χ^2^	*p* Value
Anterior temporalis muscles	36 (45.0%)	18 (22.5%)	9.06	0.003
Medial temporalis muscles	32 (40.0%)	16 (20.0%)	7.62	0.006
Posterior temporalis muscles	25 (31.3%)	12 (15.0%)	5.94	0.015
Superficial masseter muscles	58 (72.5%)	37 (46.3%)	11.43	0.001
Deep masseter muscles	59 (73.8%)	36 (45.0%)	13.71	<0.001
Medial pterygoid muscles	42 (52.5%)	40 (50.0%	0.1	0.752
Lateral pterygoid muscle	64 (80.0%)	53 (66.3%)	3.85	0.05
Digastric muscle—anterior belly	19 (23.8%)	4 (5.0%)	9.95	0.002
Digastric muscle—posterior belly	24 (30.0%)	3 (3.8%)	17.82	<0.001
Mylohyoid muscles	14 (17.7%)	5 (6.3%)	4.97	0.026
Sternocleidomastoid muscles—sternal head	60 (75.0%)	62 (77.5%)	0.138	0.71
Sternocleidomastoid muscles—clavicular head	53 (66.3%)	56 (70.0%)	0.259	0.611

**Table 7:** Oral signs for SScs and controls.

Oral Signs	SSc	Controls	χ^2^	*p* Value
Microstomia	64 (80%)	2 (2.5%)	95.96	<0.001
Oral ulcers	31 (38.8%)	9 (11.3%)	16.13	<0.001
Petechiae	18 (22.5%)	0 (0.0%)	18.09	<0.001
Gingival recession	56 (70.0%)	26 (32.5%)	22.51	<0.001
erythematous tongue	37 (46.3%)	3 (3.8%)	38.53	<0.001
Fibrous tongue	21 (26.3%)	13 (16.3%)	2.39	0.122

**Table 8:** Restricted Movements (RM).

Measurements (mm); Mean ± SD	SSc	Controls	*T* Student	*p* Value
Opening	33.29 ± 7.97	43.37 ± 6.64	−8.693	<0.001
Laterotrusion right	5.67 ± 3.14	7.3 ± 2.5	−3.622	<0.001
Laterotrusion left	5.87 ± 3.61	7.37 ±2.48	−3.059	0.009
Protrusion	4.62 ± 3.28	6.17 ± 2.6	−3.313	0.02

## Abbreviations

TMD	temporomandibular disorders
TMJ	temporomandibular joint
RDC/TMD	Research Diagnostic Criteria For Temporomandibular Disorders
TMDs	symptoms of temporomandibular disorders
OD	opening derangement
TMJs	sounds of temporomandibular joint
VAS	visual analogue scale
MP	myofascial pain
RM	restricted movements
ACR/EULAR	American College of Rheumatology and European League Against Rheumatism
